# Deep Margin Elevation With Various Resin Composites: Effect of Thermomechanical Aging on the Marginal Gap of CAD/CAM Inlays

**DOI:** 10.1111/jerd.70159

**Published:** 2026-04-08

**Authors:** Zeliha Şanıvar Abbasgholizadeh, Ezgi Göler

**Affiliations:** ^1^ Department of Prosthodontics, Faculty of Dentistry Marmara University Istanbul Türkiye

**Keywords:** adhesive interfaces, CAD/CAM inlays, deep margin elevation, marginal fit, resin composites, SEM, thermomechanical aging

## Abstract

**Objective:**

This in vitro study evaluated the effects of composite material type, margin design (deep margin elevation vs. control), and thermomechanical aging on the marginal gap of CAD/CAM inlay restorations.

**Materials and Methods:**

Standardized mesio‐occluso‐distal (MOD) inlay cavities were prepared in extracted human molars, with mesial margins located at the cemento‐enamel junction (control) and distal margins extended 2 mm apical to the CEJ and elevated using three resin composite materials (bulk‐fill, packable, and flowable). CAD/CAM inlays were fabricated and luted. Marginal gap values were measured using scanning electron microscopy at baseline and after thermomechanical aging simulating 1 year of clinical service. Data were analyzed using mixed repeated‐measures ANOVA to evaluate the effects of material type, margin design, aging condition, and their interactions (*α* = 0.05).

**Results:**

Thermomechanical aging significantly increased marginal gap values (*p* < 0.001). Margin design showed a significant effect (*p* = 0.015), whereas the main effect of material type was not statistically significant (*p* = 0.205). Significant interactions were observed between material type and margin design (*p* = 0.008) and among material type, margin design, and aging condition (*p* < 0.001).

**Conclusions:**

Within the limitations of this current study, thermomechanical aging significantly increased marginal gap values of CAD/CAM inlays. Material type alone did not show a significant main effect on marginal gap. However, significant interaction effects were observed between material type and deep margin elevation, as well as between material type and aging condition.

## 1 Introduction

Deep margin elevation (DME) is a conservative restorative technique in which subgingival proximal margins are relocated coronally using resin composites [1, 2]. This approach facilitates rubber dam isolation [3], improves impression accuracy, allows safer removal of excess cement [4], and enhances the predictability of adhesive procedures for indirect restorations [5, 6]. Favorable periodontal and restorative outcomes have been reported in both in vitro and clinical studies [6, 7, 8, 9]. However, DME remains clinically demanding due to limited access, difficulties in moisture control [3, 4], and the structural characteristics of cervical dentin that may compromise interfacial stability [10]. DME has also been described as a paradigm shift in adhesive dentistry, providing a minimally invasive alternative to surgical crown lengthening while preserving sound tooth structure [4, 5].

Although no universal consensus exists regarding the optimal restorative material for DME, resin composites remain the most widely investigated and clinically preferred option [11, 12]. A recent systematic review demonstrated their superiority over glass ionomer and resin‐modified glass ionomer materials in restoring proximal cavities with margins extending below the cemento‐enamel junction [12]. In DME procedures, resin composites are generally classified into three viscosity categories—flowable, packable, and bulk‐fill—each offering distinct handling characteristics. Flowable composites provide excellent adaptability to cavity walls but have lower filler content and reduced mechanical strength [13, 14]. Bulk‐fill composites allow placement in thicker increments, reducing operative time while maintaining adequate depth of cure and acceptable polymerization stress [15]. Packable composites, characterized by higher filler loading and greater resistance to deformation, provide stability in proximal box elevation and consistent marginal fit [16].

When DME is performed prior to indirect restoration placement, two adhesive interfaces are created: the tooth–composite and composite–restoration interfaces. The former has been widely investigated because of its biological sensitivity and susceptibility to polymerization stress, whereas the latter is equally important as it determines the marginal fit of the restoration under functional loading [17, 18, 19]. Most previous studies have focused on the tooth–composite interface, while fewer have evaluated the composite–restoration interface [20, 21].

Marginal fit plays a decisive role in the long‐term success of indirect restorations. Despite advances in adhesive systems and CAD/CAM technology, marginal gaps between restorative materials and tooth structures remain unavoidable [22, 23]. These gaps, filled with luting cement, are prone to degradation under cyclic loading, thermal fluctuation, and chemical challenges in the oral environment [24]. Previous studies have reported marginal gap values for CAD/CAM inlays and onlays ranging from 36 to 222 μm, with consistent increases following simulated aging [25]. Despite variability in reported values, marginal gap thresholds around 120 μm, and in some cases up to 200 μm, have been considered clinically acceptable for indirect restorations [26]. Artificial aging protocols have repeatedly demonstrated a decline in marginal fit, regardless of restorative material or luting agent [27, 28]. Poor marginal fit has been associated with microleakage, cement dissolution, marginal discoloration, secondary caries, and periodontal irritation [24, 27]. Multiple factors influence marginal fit, including cavity configuration, finish line design, restorative material properties, impression accuracy, and cementation technique [26, 29]. Therefore, achieving and maintaining optimal marginal fit over time remains a fundamental objective in restorative dentistry [26].

Collectively, the existing body of evidence suggests that material‐related characteristics of resin composites used for deep margin elevation may influence marginal fit at the composite–restoration interface, particularly when restorations are subjected to functional loading and thermal challenges. Variations in filler content, polymerization behavior, and resistance to deformation among flowable, packable, and bulk‐fill resin composites have been reported to affect marginal gap formation and interfacial integrity in indirect restorations [7, 8, 9, 13, 14, 15, 16]. While the tooth–composite interface has been extensively investigated because of its biological relevance, comparatively limited attention has been directed toward marginal fit at the composite–restoration interface created following margin relocation, despite its direct implications for the long‐term performance of CAD/CAM restorations [17, 18, 19, 20, 21]. In addition, thermomechanical aging has consistently been shown to increase marginal gap values in indirect restorations, underscoring the importance of aging protocols when evaluating marginal fit associated with deep margin elevation [21, 23, 24, 25]. Therefore, considering the documented influence of thermomechanical aging and material‐related characteristics on marginal integrity, evaluating the marginal fit at the composite–restoration interface following deep margin elevation appears clinically relevant. The purpose of this in vitro study was to evaluate the marginal fit at the composite–inlay interface of CAD/CAM inlay restorations placed after DME using flowable, packable, or bulk‐fill composites and to determine the effect of thermomechanical aging on marginal fit.

Based on the documented effects of composite material properties and thermomechanical aging on marginal integrity, the following hypotheses were tested within a factorial, repeated‐measures study design, including material type (flowable, packable, bulk‐fill), aging condition (baseline and thermomechanically aged), and margin design (DME and control) as study factors:

1. The marginal fit at the composite–inlay interface differs according to the type of resin composite used for deep margin elevation.
2. Thermomechanical aging affects the marginal fit of CAD/CAM inlay restorations.
3. The marginal fit of CAD/CAM inlay restorations differs between margins with and without deep margin elevation.

## 2 Materials and Methods

### 2.1 Study Design

Ethical approval for this in vitro study was obtained from the Marmara University Faculty of Medicine Ethics Committee (Approval No. 09.2023.1425). Sample size estimation was performed using G*Power software (Version 3.1; Heinrich Heine University Düsseldorf, Germany). The calculation was based on a factorial repeated‐measures design including material type, aging condition, and margin design as study factors. Assumptions regarding expected differences in marginal gap values after thermomechanical aging were informed by previously published in vitro studies with similar experimental protocols [25]. With a significance level of 0.05 and a desired statistical power of 0.80, a total sample size of 30 extracted human molars (*n* = 10 per material group) was determined to be sufficient for detecting relevant effects and interactions among the study factors.

### 2.2 Specimen Preparation

Thirty noncarious, unrestored human molars were collected from the Department of Oral and Maxillofacial Surgery, Marmara University Faculty of Dentistry. Residual soft tissues and deposits were removed, and the teeth were stored in distilled water, refreshed weekly. Each tooth was embedded in cold‐cured acrylic resin with the cemento‐enamel junction (CEJ) positioned 3 mm above the resin surface. Teeth extracted for periodontal or orthodontic reasons were included, and only structurally intact, noncarious, unrestored molars without cracks, wear, or root resorption were selected.

Standardized mesio‐occluso‐distal (MOD) inlay cavities were prepared under water cooling. Cavity preparations were performed using cylindrical diamond burs (Hager & Meisinger, Neuss, Germany), which were replaced after every three preparations to maintain consistent cutting efficiency and surface quality. The occlusal cavity width corresponded to one‐third of the buccolingual cusp distance, and the proximal box width was one‐third of the buccolingual width at the proximal surface. The distal box extended 2 mm apical to the CEJ, while the mesial box terminated at the CEJ. Cavities had a mesiodistal width of 2 mm and a buccolingual width of 5 mm. All internal angles were rounded, and no bevels were prepared.

### 2.3 Group Allocation and Deep Margin Elevation

Specimens were randomly divided into three groups (*n* = 10) based on the composite material used for DME at the distal margin:

- Group 1: Bulk‐fill composite (Venus Bulk Fill, Kulzer, Hanau, Germany)
- Group 2: Packable composite (*G‐*aenial A'CHORD, GC, Tokyo, Japan)
- Group 3: Flowable composite (G‐aenial Universal Injectable, GC, Tokyo, Japan)

The resin matrix composition, filler characteristics, and filler load of the composite materials used for deep margin elevation are summarized in Table 1.

**TABLE 1:** Composition and filler characteristics of the resin composites used for deep margin elevation.

Material	Composite category	Manufacturer	Matrix composition	Filler type/characteristics	Filler size (μm/nm)	Filler load (wt%)	Filler load (vol%)
Venus Bulk Fill	Bulk‐fill flowable composite	Kulzer, Hanau, Germany	Methacrylate‐based resin matrix	Nano‐hybrid inorganic fillers (barium glass and silica); radiopaque	Nano‐scale (exact size not specified)	65	41
G‐ænial A'CHORD	Universal resin composite	GC Corporation, Tokyo, Japan	Methacrylate‐based resin matrix (Bis‐MEPP monomer)	Barium glass‐based nano‐hybrid filler with FulL Coverage Silane Coating and High‐Performance Pulverized CERASMART technology; radiopaque	≈300 nm	82	70.2
G‐ænial Universal Injectable	Injectable/flowable composite	GC Corporation, Tokyo, Japan	Methacrylate monomer (31 wt%)	Ultra‐fine barium glass and silica fillers; nano‐hybrid; radiopaque	≈150 nm	69	50

*Note:* Data based on manufacturers' technical information.

Abbreviations: ≈, approximate values; vol%, volume percentage; wt%, weight percentage.

The mesial margins were not subjected to DME and served as control regions.

**FIGURE 1:**
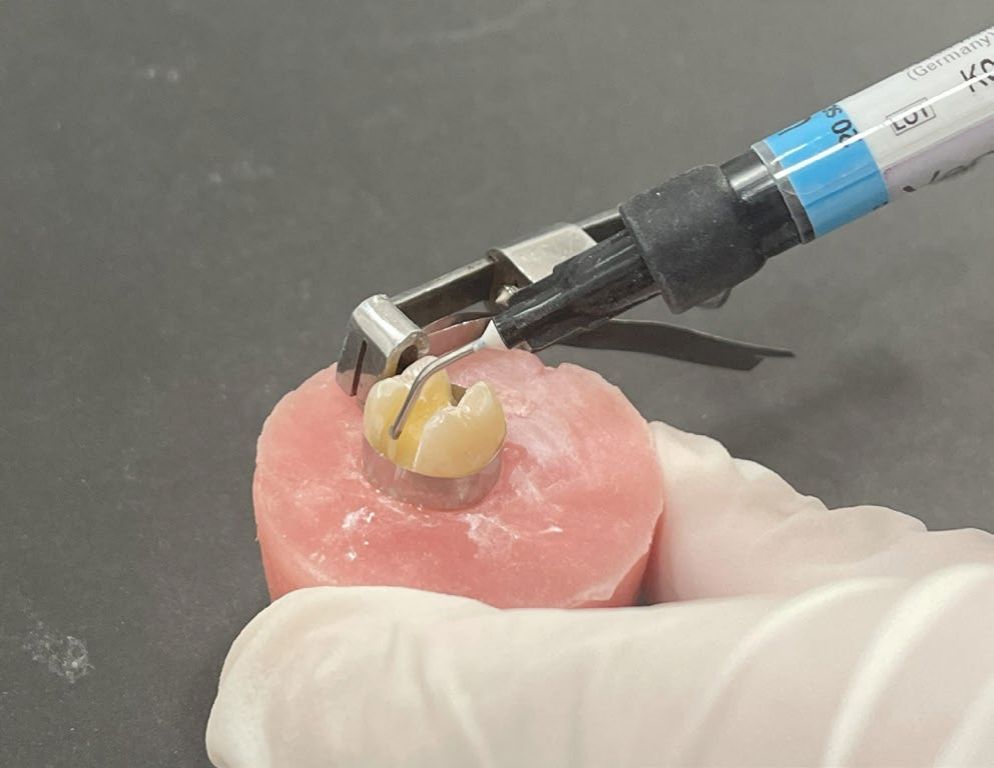
Schematic representation of deep margin elevation (DME) performed at the distal margin using composite applied in 1‐mm increments, followed by light curing of each layer.

DME was performed using a Tofflemire matrix band trimmed to 2 mm height. After matrix placement, enamel margins were selectively etched with 37% phosphoric acid for 15 s, while dentin surfaces were left unetched. A universal adhesive (G‐Premio Bond, GC, Tokyo, Japan) was applied in self‐etch mode and light‐cured using an LED curing unit (Elipar DeepCure‐S, 3 M, Seefeld, Germany). Composite layers were placed in 1‐mm increments (Figure 1) and light‐cured for 20 s per layer, with the light output verified before each session using a radiometer (> 1000 mW/cm^2^). The total thickness of the composite used for deep margin elevation was standardized at 2 mm for all specimens. Residual composite excesses were finished with a fine‐grit diamond bur (Hager & Meisinger, Neuss, Germany). All procedures were performed by a single experienced operator to ensure consistency, and randomization was conducted using computer‐generated random numbers.

### 2.4 Fabrication of CAD/CAM Restorations

**FIGURE 2:**
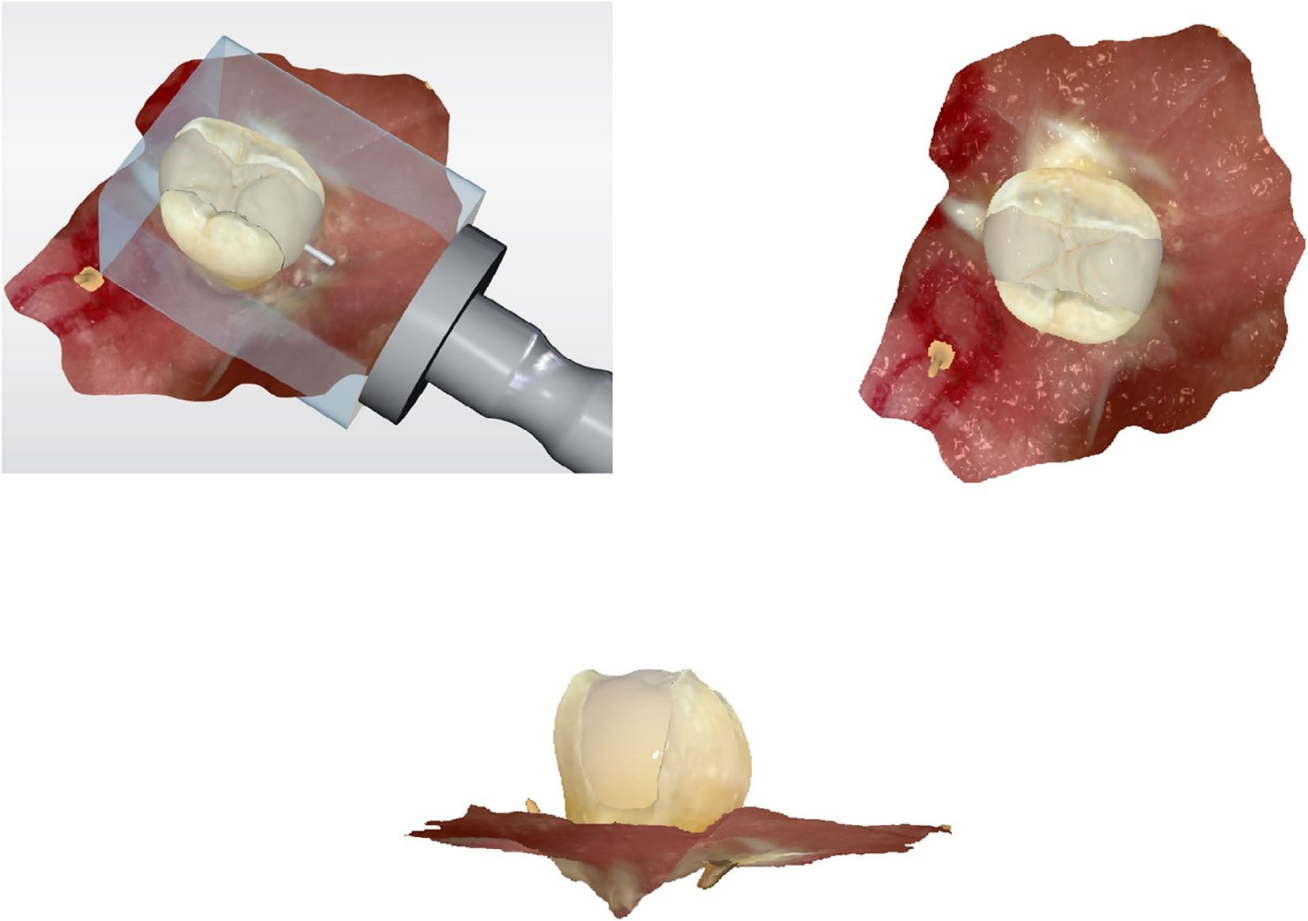
Digital scanning, virtual design, and CAD/CAM fabrication workflow of the MOD inlay restorations.

All MOD cavities were scanned with an intraoral scanner (CEREC AC, Dentsply Sirona, Bensheim, Germany), and the restorations were milled from a resin nano‐ceramic block (CERASMART 270, GC, Tokyo, Japan) (Figure 2).

### 2.5 Luting Procedure

The internal surface of each restoration was air‐abraded with 30‐μm silica particles (CoJet System, 3 M, Seefeld, Germany) at 2.5 bar pressure and then rinsed. Selective enamel etching was performed with 37% phosphoric acid gel (FineEtch, Spident, Incheon, South Korea) for 15 s. The restoration surface at the distal step was treated with a silane coupling agent (3 M Silane, 3 M, St. Paul, USA) and gently dried.

A universal adhesive (G‐Premio Bond, GC, Tokyo, Japan) was applied, air‐dried, and light‐cured. Prior to cementation, the internal adaptation and seating of each restoration were verified using a low‐viscosity silicone material (Elite HD+, Zhermack, Italy) to ensure complete seating and detect any internal interferences. All restorations were cemented using a dual‐cure resin cement (G‐CEM LinkForce, GC, Tokyo, Japan). Excess cement was removed with a #15c scalpel blade, and all specimens were stored in distilled water for 24 h before replica preparation.

### 2.6 Epoxy Replica Technique and SEM Analysis

**FIGURE 3:**
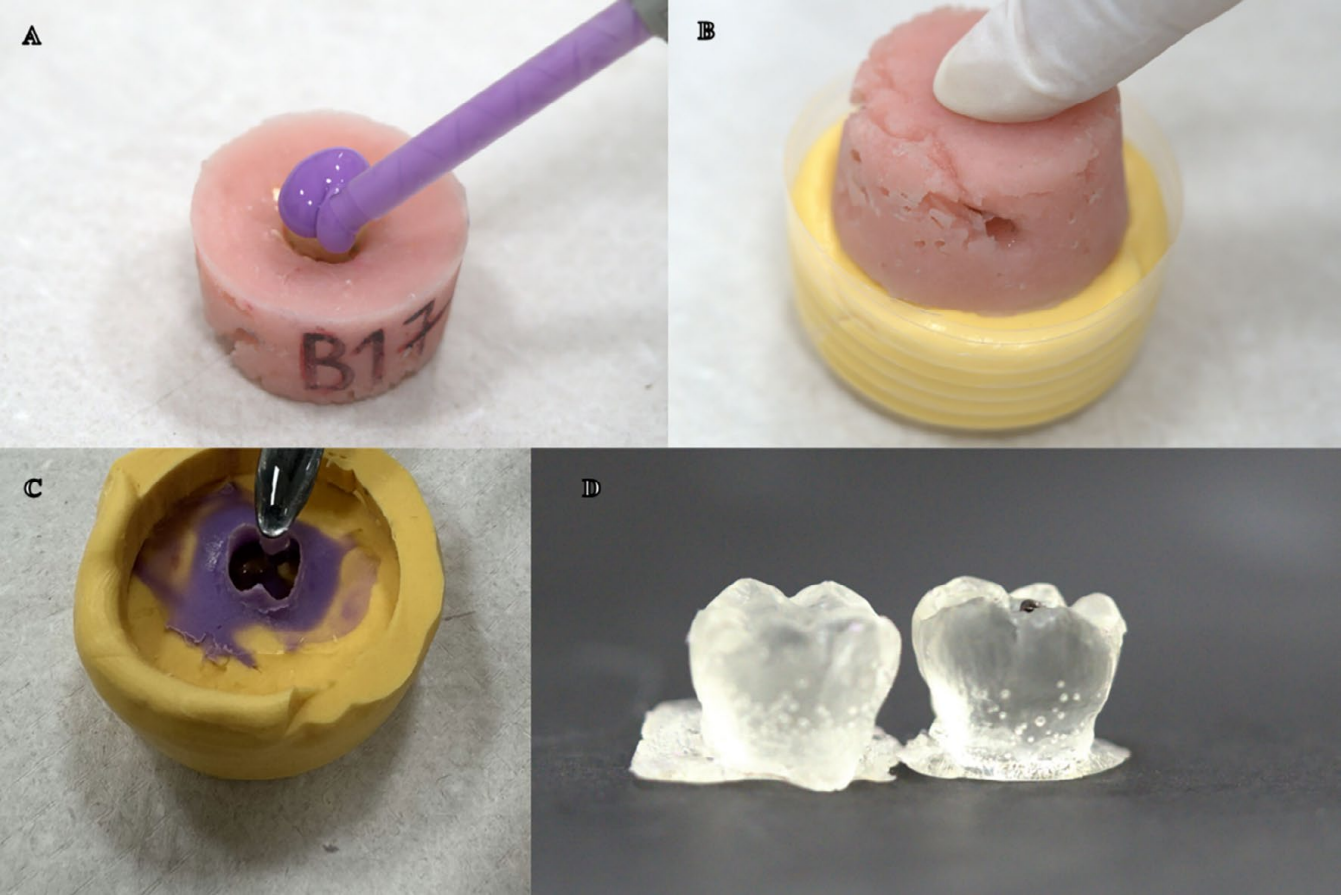
Workflow of the epoxy replica technique used for marginal gap evaluation. (A) Application of light‐body silicone around the restoration to capture marginal details. (B) Stabilization of the impression using heavy‐body silicone in a custom mold. (C) Pouring of low‐viscosity epoxy resin into the silicone mold to obtain replicas. (D) Representative epoxy resin replicas of the specimens prior to sectioning for SEM analysis.

Marginal impressions were obtained both before and after thermomechanical aging using light‐ and heavy‐body silicone materials (Elite HD+, Zhermack, Badia Polesine, Italy). A light‐body silicone was applied around the restoration to record marginal details, followed by a heavy‐body silicone in a custom mold for stabilization. After polymerization, a low‐viscosity epoxy resin (Epofix, Struers, Ballerup, Denmark) was poured into the molds to obtain replicas for marginal gap measurements. The epoxy was cured for 24 h at room temperature before sectioning (Figure 3). During replica fabrication, particular attention was paid to avoiding air bubble formation, and only defect‐free marginal regions were selected for SEM evaluation.

**FIGURE 4:**
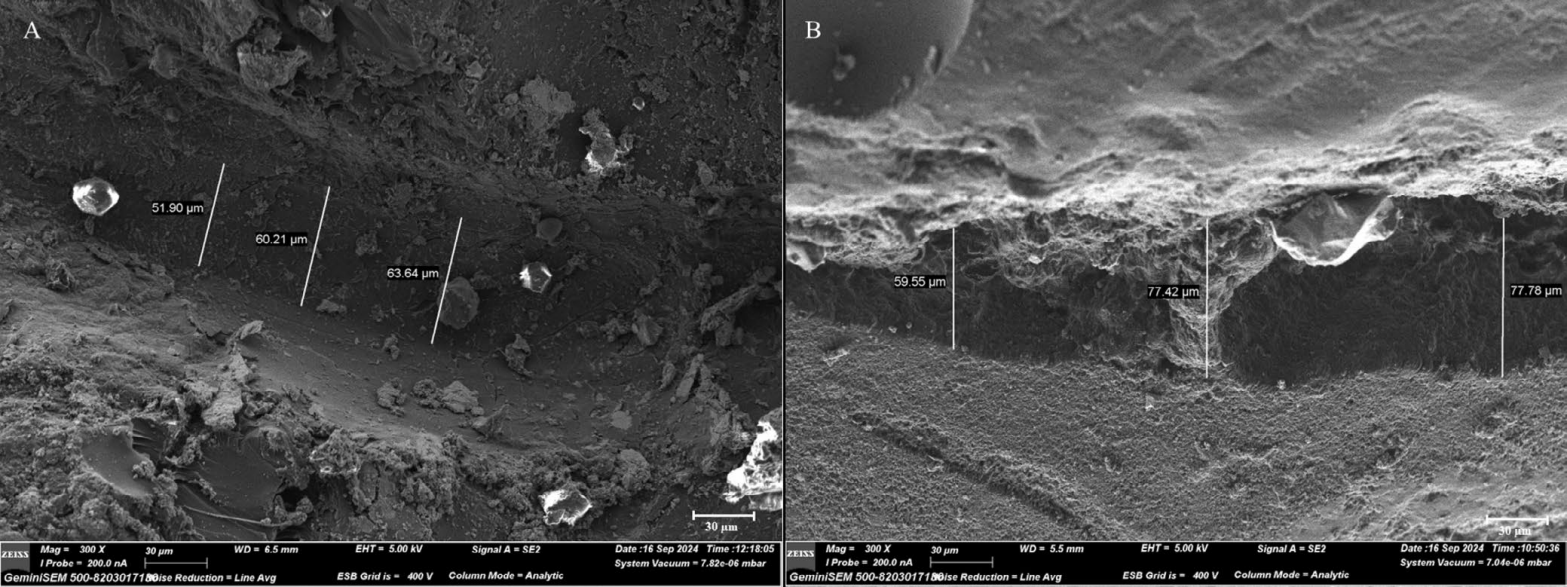
Representative SEM micrographs of marginal fit at baseline (A) and after thermomechanical aging (B). Vertical lines indicate representative marginal gap measurements (μm). Images were obtained at ×300 magnification. Scale bar = 30 μm.

Each epoxy model was sectioned buccolingually and divided into mesial and distal halves. The replicas were sputter‐coated with a gold–palladium layer (~10 nm) using a sputter coater (Quorum SC 7620, Quorum Technologies, Sussex, UK). Marginal gap measurements were performed under a scanning electron microscope (SEM) (GeminiSEM 500, Carl Zeiss AG, Jena, Germany) at ×300 magnification. Representative SEM micrographs of the marginal interface obtained before (Figure 4A) and after thermomechanical aging (Figure 4B) were used for evaluation. Eight equidistant measurement points were recorded along each margin. For each margin, the mean value of the eight measurements was calculated and used for statistical analysis. The epoxy replica technique is a well‐established and validated method for non‐destructive marginal gap evaluation [30]. Measurements were performed by two blinded examiners, and 10 specimens were re‐evaluated after 1 week to assess intraexaminer reliability (> 90% agreement).

### 2.7 Thermomechanical Aging

All specimens underwent thermomechanical aging using a dual‐axis chewing simulator (CS‐4, SD Mechatronik, Feldkirchen‐Westerham, Germany). The protocol included 240,000 mechanical loading cycles at 50 N with a 1‐mm round‐ended stainless‐steel tip applied vertically at the central fossa. Additionally, 2400 thermal cycles were performed between 5°C and 55°C, with a 30‐s dwell time, simulating approximately 1 year of clinical function. All thermal cycles were performed in distilled water to simulate the oral environment.

### 2.8 Statistical Analysis

Statistical analyses were performed using SPSS software (Version 23.0; IBM, Chicago, USA). The normality of the data distribution was assessed using the Shapiro–Wilk test. A mixed repeated‐measures ANOVA was specified to evaluate the effects of material, aging condition, and margin design on the outcome variable. Material (three levels) was defined as the between‐subject factor, whereas aging condition (baseline vs. post‐aging) and margin design (mesial vs. distal) were defined as within‐subject factors. Main effects and all two‐way and three‐way interactions (material × aging, material × margin, aging × margin, and material × aging × margin) were examined. Model assumptions were examined, and appropriate corrections were applied when necessary. Post hoc pairwise comparisons were performed with Bonferroni adjustment for multiple testing.

The experimental workflow and all measurements followed ISO 11405 and ASTM E1951 guidelines where applicable. No generative artificial intelligence tools (such as ChatGPT or other large language model‐based assistants) were used to generate, analyze, or edit the scientific content of this manuscript. All wording, data interpretation, and conclusions were created solely by the authors.

## 3 Results

Descriptive statistics of marginal gap values according to material group, margin design, and aging condition are presented in Table 2.

**TABLE 2:** Descriptive statistics of marginal gap values (μm) according to material group, margin design, and aging condition.

Material group	Margin	Baseline (mean ± SD)	Post‐aging (mean ± SD)
B (Bulk‐fill)	Mesial	60.1 ± 5.2	71.4 ± 5.5
	Distal	58.1 ± 4.2	66.7 ± 3.9
P (Packable)	Mesial	62.1 ± 5.3	73.3 ± 5.8
	Distal	57.5 ± 5.9	66.1 ± 3.8
F (Flowable)	Mesial	59.1 ± 6.1	64.3 ± 6.1
	Distal	58.4 ± 5.2	68.6 ± 5.1

*Note:* Values are presented as mean ± standard deviation (μm). Baseline measurements were obtained before thermomechanical aging, and post‐aging measurements were obtained after aging.

Abbreviations: B, bulk‐fill resin composite; F, flowable resin composite; P, packable resin composite.

The mixed repeated‐measures ANOVA revealed a statistically significant three‐way interaction among material group, margin design, and aging condition (Table 3).

**TABLE 3:** Results of mixed repeated‐measures ANOVA for the effects of material group, margin design, aging condition, and their interactions on marginal gap values.

Effect	*F*(df1, df2)	*p*	Partial *η* ^2^
Material Group	*F*(2, 54) = 1.63	0.205	0.05
Margin Design	*F*(1, 54) = 6.28	0.015	0.10
Aging Condition	*F*(1, 54) = 504.46	< 0.001	0.90
Material Group × Margin Design	*F*(2, 54) = 5.29	0.008	0.16
Material Group × Aging Condition	*F*(2, 54) = 3.07	0.055	0.10
Margin Design × Aging Condition	*F*(1, 54) = 0.00	0.935	0.00
Material Group × Margin Design × Aging Condition	*F*(2, 54) = 9.24	< 0.001	0.25

*Note:* Results obtained from mixed repeated‐measures ANOVA. Partial *η* ^2^ represents effect size. Statistical significance was set at *α* = 0.05.

Among the two‐way interactions, a significant interaction was observed between material group and margin design, whereas the interactions between material group and aging condition and between margin design and aging condition were not statistically significant (Table 3).

Regarding the main effects, aging condition and margin design showed significant effects on marginal gap values, whereas the main effect of material group was not statistically significant (Table 3).

Bonferroni‐adjusted pairwise comparisons based on estimated marginal means indicated that marginal gap values were significantly higher after thermomechanical aging compared with baseline. Additionally, mesial margins exhibited significantly greater marginal gap values than distal margins (Table 4).

**TABLE 4:** Bonferroni‐adjusted pairwise comparisons for the main effects of aging condition and margin design on marginal gap values.

Comparison	Mean difference (μm)	95% CI	*p*
Baseline vs. Post‐Aging	9.2	8.4 to 10.0	< 0.001
Mesial vs. Distal	2.4	0.50 to 4.4	0.015

*Note:* Pairwise comparisons were based on estimated marginal means with Bonferroni adjustment for multiple testing.

## 4 Discussion

This study evaluated the marginal fit at the composite–inlay interface after deep margin elevation (DME) using flowable, packable, and bulk‐fill composites and assessed the influence of thermomechanical aging and margin design. This study showed that the composite category did not significantly influence marginal fit, whereas thermomechanical aging and margin design significantly affected marginal fit. Moreover, the significant interaction effects observed in the factorial repeated‐measures model indicate that the influence of composite material on marginal fit depends on the margin configuration and aging condition rather than functioning as an independent determinant.

The findings did not demonstrate a significant main effect of composite material type on marginal fit, as no significant differences were observed among flowable, packable, and bulk‐fill groups before or after aging. Comparable outcomes have been described when standardized adhesive protocols and incremental layering are applied, yielding similar marginal adaptation across different composite viscosities [18, 21, 22]. The absence of intergroup differences in the present work is consistent with this rationale, as all materials were placed in 1‐mm increments to reduce polymerization stress and promote interfacial continuity. The flowable composite used in this study was a manufacturer‐classified highly filled material designed for injection techniques and therefore does not represent a conventional low‐viscosity formulation. However, the significant interaction between material type and margin design observed in the factorial model suggests that material‐related effects depend on the clinical configuration of the margin and the aging condition.

Evidence supports the preference for resin composites over glass ionomer–based materials for margin relocation [7, 9, 20]. Improved marginal integrity relative to resin‐modified or conventional glass ionomers has been documented, reinforcing the reliability of resin composites for subgingival elevation [7, 9]. Substrate‐dependent behavior has also been noted; packable composites tend to perform favorably at enamel margins, whereas flowable bulk‐fills have been reported to show advantages at dentin margins [9, 22]. Because margins in the present study were primarily located in dentin, the similarity among composite categories aligns with these observations. However, considering the significant interaction between material type and margin design observed in the present study, the influence of composite category on marginal fit should be interpreted as configuration‐dependent rather than as universally equivalent across all clinical conditions.

Overall, restorative technique and adherence to adhesive protocols appear to exert a greater influence on marginal adaptation than intrinsic material viscosity. Nevertheless, the interaction findings indicate that the contribution of material type cannot be considered entirely independent, as its effect depends on margin configuration and aging condition. The comparable marginal fit across all categories in this study supports the clinical flexibility of DME in varied restorative scenarios, while emphasizing that material‐related effects should be interpreted within the specific clinical context rather than in isolation. Regarding the effect of thermomechanical aging on marginal fit, the findings of the present study demonstrated a significant increase in marginal gap values after aging. All groups exhibited increased marginal gap values after thermomechanical aging, indicating that cyclic loading and thermal stress compromised interfacial integrity, although the extent of this effect was influenced by margin design and composite material. Increased gap formation and reduced marginal continuity following artificial aging have been consistently shown [15, 21, 23, 24, 27]. Aging simulation therefore remains essential for realistic appraisal of long‐term adhesive performance. However, the post‐aging marginal gap values observed in this study remained within the ranges reported as clinically acceptable for indirect restorations.

Thermomechanical cycling has been associated with a reduction in continuous margins for indirect restorations, with elevated and non‐elevated margins similarly affected [8, 21, 25]. The consistency of this effect across materials and strategies suggests degradation driven primarily by mechanical fatigue although the extent of this degradation was influenced by material characteristics and margin configuration. Fatigue may promote microcrack propagation and stress concentration at the cement interface, widening marginal gaps over time.

The aging regimen used here—240,000 mechanical cycles and 2400 thermal cycles between 5°C and 55°C—was selected to approximate 1 year of clinical function and has been widely adopted [21, 23, 24]. This standardized simulation protocol provides a clinically relevant framework for interpreting the observed increase in marginal gap values after aging. Although longer simulations could yield additional insight, the present protocol enables standardized comparison with existing literature under clinically meaningful conditions.

Mean marginal gap values were calculated from eight equidistant measurement points along each margin, providing a representative evaluation of marginal fit while reducing the influence of localized variability. This metric has been applied previously and has been considered clinically relevant and reproducible [7, 15, 20, 26]. In the context of the present findings, evaluation of the mean gap enabled a clearer interpretation of the effects of thermomechanical aging and margin configuration on marginal fit. Although mean‐based or percentage‐of‐continuous‐margins approaches are also used, the use of mean values allows a reliable assessment of overall marginal fit under functional loading conditions.

The third hypothesis examined whether marginal fit differed between margins with and without deep margin elevation. The present findings demonstrated that margin design significantly influenced marginal fit. Mesial control margins exhibited higher marginal gap values than distal margins relocated coronally with resin composite. This pattern indicates that coronal relocation of deep proximal margins does not compromise the marginal fit of CAD/CAM inlays when standardized adhesive protocols are applied [1, 23, 28], while the significant interaction effects observed in the factorial analysis suggest that the influence of margin design is not independent but depends on composite material and aging condition. Accordingly, the effect of deep margin elevation on marginal fit should be interpreted as material‐ and aging‐dependent rather than as a uniform phenomenon. From a clinical perspective, these findings support the use of deep margin elevation as a conservative restorative approach for managing deep subgingival margins while maintaining satisfactory marginal fit.

Collectively, the present findings indicate that thermomechanical aging adversely affected marginal fit, and that the magnitude of this effect was influenced by margin design and composite material, as reflected by the observed interaction effects. These results highlight the importance of incorporating fatigue simulation when evaluating the long‐term behavior of adhesive interfaces, as short‐term laboratory assessments may not fully capture material‐ and design‐dependent changes over time. In this regard, deep margin elevation performed with flowable, packable, or bulk‐fill composites yielded marginal gap values that may be considered clinically acceptable within the limitations of this in vitro study.

Nevertheless, several limitations should be considered when interpreting the generalizability of the present findings. The applied loading protocol, consisting of a vertical load directed to the central fossa, together with the standardized tooth geometry and antagonist morphology, may not fully replicate the complex proximal stress distribution encountered clinically. In addition, all specimen preparation and restorative procedures were performed by a single operator, which, although ensuring procedural standardization, may introduce operator‐related bias. Variability among extracted teeth, including potential differences in donor age and degree of dentin sclerosis, was not stratified or controlled despite the selection of intact, noncarious molars. Furthermore, inherent methodological limitations of the replica technique, particularly the risk of impression distortion during silicone replication, should be acknowledged.

## 5 Conclusion

Within the limitations of this current study, it was concluded that:

1. The marginal fit at the composite–inlay interface did not differ significantly according to the type of resin composite used for deep margin elevation using an incremental layering technique.
2. Thermomechanical aging significantly affected the marginal fit of computer‐aided design/computer‐aided manufacturing inlay restorations.
3. Marginal fit differed significantly between margins with and without deep margin elevation; however, the direction and magnitude of these differences varied according to composite material and aging condition.

## Funding

The authors have nothing to report.

## Disclosure

The authors declare no financial interest related to this study. The CAD/CAM blocks used in this investigation were kindly provided by GC Türkiye.

## Conflicts of Interest

The authors declare no conflicts of interest.

## Data Availability

The data that support the findings of this study are available from the corresponding author upon reasonable request.
